# Molecular Mechanisms Underlying Alzheimer’s Disease Pathogenesis: Comprehensive Overview

**DOI:** 10.3390/ijms27104651

**Published:** 2026-05-21

**Authors:** Filomena Lo Vecchio, Annamaria la Torre, Carolina Gravina, Grazia D’Onofrio, Antonio Greco

**Affiliations:** 1 Laboratory of Gerontology and Geriatrics, Fondazione IRCCS Casa Sollievo Della Sofferenza, San Giovanni Rotondo, 71013 Foggia, Italy; f.lovecchio@operapadrepio.it (F.L.V.); cgravina@operapadrepio.it (C.G.); 2 Clinical Psychology Service, Health Department, Fondazione IRCCS Casa Sollievo Della Sofferenza, San Giovanni Rotondo, 71013 Foggia, Italy; g.donofrio@operapadrepio.it; 3 Complex Unit of Geriatrics, Department of Medical Sciences, Fondazione IRCCS Casa Sollievo Della Sofferenza, San Giovanni Rotondo, 71013 Foggia, Italy; a.greco@operapadrepio.it

**Keywords:** Alzheimer’s disease, β-amyloid, neurofibrillary tangles, autophagy, mitochondrial dysfunction, stress response

## Abstract

Alzheimer’s disease (AD) is a progressive, multifactorial neurodegenerative disorder ranking first as cause of dementia in the elderly. It is characterized by the progressive deterioration of the central nervous system, leading to impaired cognitive function and reduced ability to perform daily activities. Pathological hallmarks of AD include the accumulation of β-amyloid plaques and neurofibrillary tangles which ultimately cause neuronal death and synaptic loss. The vast majority of AD cases are sporadic, with aging representing the primary non-modifiable risk factor contributing to disease susceptibility and progression. However, several factors encompassing genetic predisposition, systemic inflammation, chronic diseases, infections, traumatic brain injury, lifestyle factors, and environmental exposures may affect AD onset. This work aims to describe and discuss the main molecular pathways involved in AD pathophysiology and to examine how these mechanisms cross-interact in promoting neurodegeneration and disease progression.

## 1. Introduction

Alzheimer’s disease (AD) is the major form of neurodegenerative disorder, accounting for 60–80% of dementia cases worldwide [1]. The key pathological changes include the accumulation of extracellular β-amyloid plaques (Aβ) and intracellular aggregates of hyperphosphorylated tau protein that form neurofibrillary tangles (NFTs), resulting in the progressive degeneration of neurons and the related loss of specific synaptic communication [2]. Brain regions such as the hippocampus and cortex are particularly susceptible to amyloid deposition and NFTs [3].

The core clinical features of AD consist of progressive memory loss, cognitive decline, functional impairment, and behavioral changes [4] that culminate in long-term disability and death [5]. AD incidence and prevalence increase dramatically with age, with women carrying a higher risk of developing the disease than men [1]. Initially, AD pathology may be asymptomatic, first manifesting as mild cognitive impairment (MCI; prodromal phase) before progressing to overt dementia (dementia phase) [2].

AD may be broadly classified into two forms. Familial AD follows an autosomal dominant pattern of inheritance and typically presents as early-onset AD (EOAD), occurring before the age of 65 years and accounting for approximately 1–5% of cases. It is caused by pathogenic mutations in specific genes, including presenilin-1 gene (*PSEN1* OMIM #104311, 14q24.2), presenilin-2 gene (*PSEN2* OMIM #600759, 1q42.13), and amyloid precursor protein (*APP* OMIM #104760, 21q21.3). In contrast, late-onset AD (LOAD), which develops after 65 years of age, represents approximately 95% of cases. Despite age being the principal risk factor for LOAD [6], several additional risk factors have been identified, including female sex, traumatic brain injury, depression, unhealthy diet, environmental pollution, physical inactivity, social isolation, low educational attainment, metabolic syndrome, and genetic susceptibility, particularly variants in the ε4 allele of apolipoprotein E. (*APOE* OMIM #107741, 19q13.32) [7]. Indeed, the presence of one or two *APOE* ε4 (*APOE4*) alleles confers an estimated 3–4-fold or 9–15-fold increased risk of developing AD, respectively [2,8,9,10].

In the clinical setting, current strategies for AD diagnosis may combine both invasive and minimally invasive biomarkers. The combination of cerebrospinal fluid (CSF) and imaging (PET) biomarkers, by measuring Aβ42 concentration, Aβ42/Aβ40 ratio, phosphorylated tau (p-tau), total tau (t-tau), p-tau ratios, neurofilaments, synaptic proteins, inflammatory markers, and indicators of astrocyte activation, have demonstrated to ameliorate the accuracy of clinical diagnostics of AD. However, AD PET and CSF AD biomarkers are not widely available, as they are either very costly (PET) or invasive (CSF). Rather, the implementation of blood-based biomarkers as well as other biological fluids such as saliva hold the potential to become easily available and cost-effective tools for improving the diagnostic workup of AD globally [11].

Thus, understanding the molecular mechanisms underlying AD pathophysiology is crucial for the development of disease-modifying therapies and monitoring of their downstream effects as well as for the identification of potential therapeutic targets [12].

## 2. The Molecular Mechanisms in Alzheimer’s Disease

Among the molecular processes that drive neurodegeneration, such as neuroinflammation, oxidative stress, lipid and metabolic dysregulation, neurovascular dysfunction, and abnormal protein folding, shedding, and diffusion, the deposition of Aβ and the hyperphosphorylated tau protein play a central role [13].

### 2.1. Amyloid Beta Peptide (Aβ) Structure and Functions

Aβ is a short 4.2 kDa peptide consisting of 40–42 amino acids [14] and it is the principal component of the Aβ plaques. Aβ is generated through the proteolytic processing of the amyloid precursor protein (APP), a type I transmembrane protein that is expressed in many tissues [15] and enriched at neuronal synapses [14]. Its structure includes a large extracellular amino (N)-terminal domain, a hydrophobic transmembrane domain, and a short intracellular carboxyl (C)-terminal domain [2,11]. It may exist in three major isoforms (i.e., APP695, AAP751 and APP770) that originate through alternative splicing of the *APP* gene, located on human chromosome 21 and containing 18 exons [2]. In particular, APP695 is predominantly expressed in neurons, whereas APP751 and APP770 are expressed in both neurons and glial cells [16]. Once synthesized in the endoplasmic reticulum (ER), APP is transported to the Golgi apparatus to complete its maturation. From the Golgi, APP is trafficked to the plasma membrane via trans-Golgi network (TGN) derived secretory vesicles [17,18] where it can be sequentially cleaved through two alternative pathways that are in dynamic equilibrium: the amyloidogenic pathway, which leads to the production of neurotoxic Aβ peptides, and the non-amyloidogenic pathway, which prevents Aβ formation [19].

**Figure 1:**
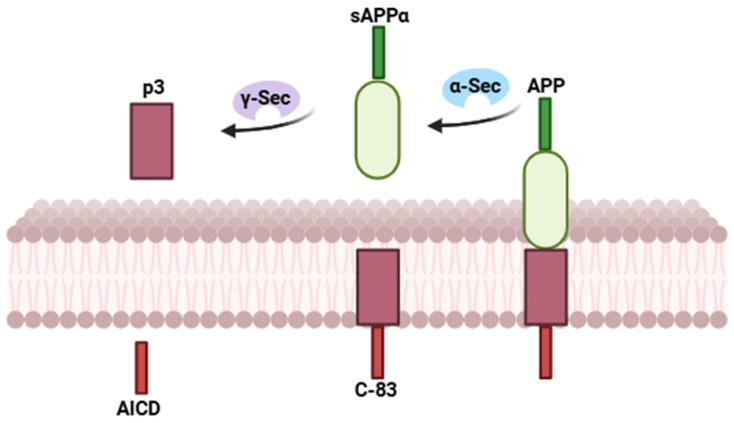
Non-amyloidogenic pathway: the amyloid precursor protein (APP) is cleaved by α-secretase within the Aβ domain, preventing the formation of Aβ peptides and releasing the soluble ectodomain sAPPα. The remaining C83 fragment is subsequently processed by γ-secretase, generating the p3 peptide and the APP intracellular domain (AICD). This pathway precludes amyloid-β production and is considered neuroprotective.

In the non-amyloidogenic pathway (Figure 1), the α-secretase-mediated cleavage of APP generates the soluble N-terminal fragment sAPPα and the membrane-bound C-terminal fragment C83. Enzymes with α-secretase activity belong to the a disintegrin and metalloproteinase (ADAM) family, including ADAM9, ADAM10, and ADAM17 [16,20]. C83 is subsequently cleaved by the γ-secretase complex, composed of presenilin 1, PSEN-2, APH-1, and nicastrin [16], that produces a p3 peptide and the APP intracellular domain (AICD) [16,21,22].

**Figure 2:**
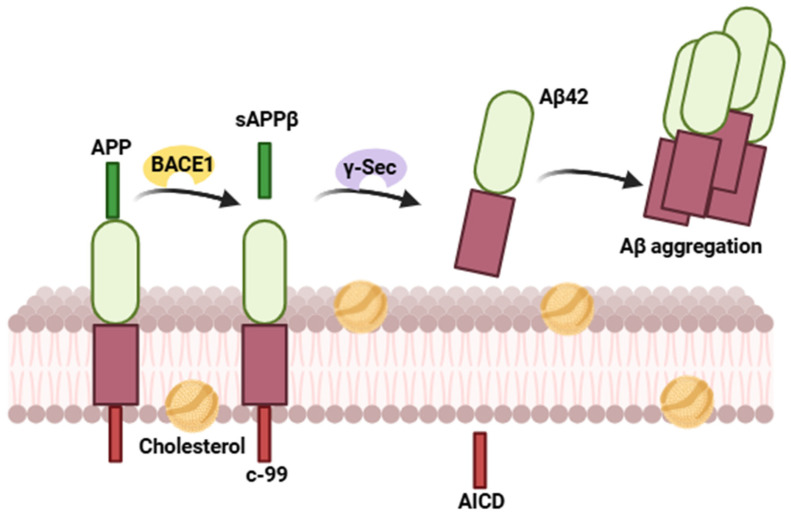
Amyloidogenic pathway: the amyloid precursor protein (APP) is first cleaved by β-secretase, generating the C99 fragment, which is subsequently processed by γ-secretase to release Aβ peptides. These peptides tend to aggregate, forming oligomers and amyloid plaques, hallmark pathological features of Alzheimer’s disease.

In the amyloidogenic pathway (Figure 2), APP is cleaved by the β-secretase or β-site APP cleaving enzyme 1 (BACE1), mainly in endosomes and lysosomes. BACE1 cleaves the isoform APP695 at Asp1 between Met596 and Asp597 [10], producing the soluble N-terminal APP fragment (sAPPβ), which is released into the extracellular space, and the C-terminal fragment C99 that remains associated with the plasma membrane. Subsequently, C99 is processed by the γ-secretase complex, resulting in the release of Aβ and the AICD. Within these lipid rafts, Aβ interacts with the ganglioside GM1, promoting its aggregation [15], whereas AICD can translocate to the nucleus, where it participates in the regulation of gene expression [23].

To maintain Aβ homeostasis, it is necessary to prevent its toxic aggregation into misfolded aggregates by maintaining a balance between Aβ generation and clearance [24] mainly mediated by zinc metalloproteinase such as neprilysin [10].

**Figure 3:**
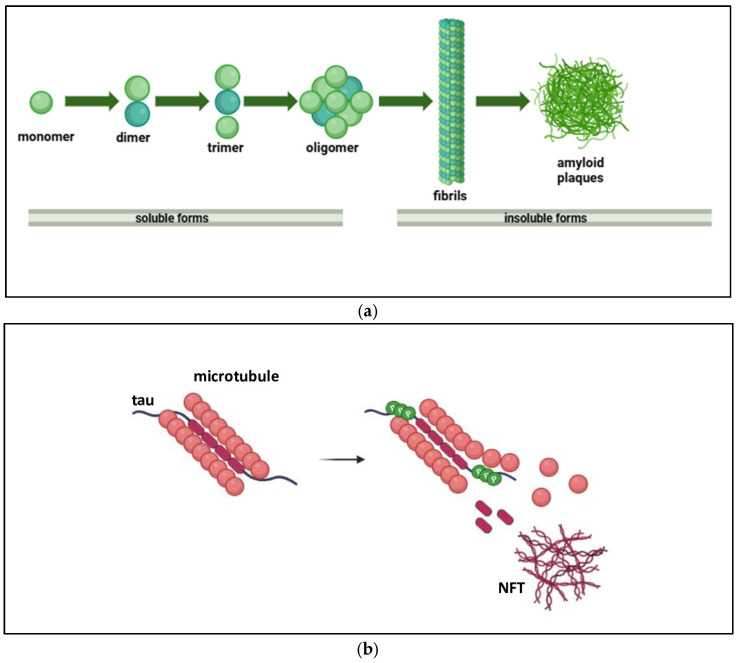
(**a**) Formation of amyloid plaques: Aβ peptides, generated from the sequential cleavage of APP, initially exist as soluble monomers that misfold and aggregate into soluble oligomers and protofibrils, which are considered highly neurotoxic. These species further assemble into insoluble fibrils that accumulate extracellularly, forming dense-core amyloid plaques. Thus, Aβ aggregation involves a dynamic transition from soluble forms (monomers, oligomers, protofibrils) to insoluble fibrillar deposits, a key pathological hallmark of AD. (**b**) Formation of neurofibrillary tangles: the microtubule-associated protein tau undergoes abnormal hyperphosphorylation, leading to its detachment from microtubules and loss of normal stabilizing function. Misfolded tau aggregates into soluble oligomeric species, which further assemble into paired helical filaments and straight filaments. These insoluble fibrillar structures accumulate intracellularly as neurofibrillary tangles, a key pathological hallmark of AD associated with neuronal dysfunction and degeneration.

However, under pathological conditions, APP is preferentially processed through the amyloidogenic pathway, leading to the accumulation of Aβ in the brain [23]. The overproduction and abnormal accumulation of Aβ ultimately results in cytotoxicity and synaptotoxicity, triggering subsequent neurodegenerative processes including synaptic dysfunction, formation of NFTs and neuron loss, which ultimately lead to neuronal death [16]. Amyloid deposition begins in the isocortex and subsequently extends to the allocortex and subcortical structures such as the basal ganglia and brainstem [13,19]. Numerous different Aβ species exist, but two main toxic forms of Aβ are produced: Aβ1-40 and Aβ1-42. These are intrinsically disordered proteins (IDPs), with a molecular weight of approximately 4.3 kDa and 4.5 kDa and isoelectric points (pI) of about 5.4 and 5.5, respectively [11]. The relative amount of Aβ1-42 is particularly higher within amyloid plaques than Aβ1-40 because it is more prone to aggregation due to its higher fibrillization rate and lower solubility compared with Aβ1-40 [25]. Indeed, through their different propensities for misfolding and self-assembly, the conformational heterogeneity of Aβ1-42 fibrils compared to Aβ1-40 fibrils has been identified. In Aβ1-40 fibrils, the monomeric units can be arranged with double or triple symmetry, whereas in Aβ1-42 fibrils only a double symmetry configuration is observed. Thus, the addition of two amino acids to the C-terminus of Aβ1-42 promotes the formation of a longer hydrophobic cluster during aggregation, resulting in rigid fibrillar core structures that differ from those of Aβ1-40 fibrils [26]. These higher-order oligomers ultimately develop into protofibrils and amyloid fibrils, which are readily detectable in AD brains [15,27,28]. Amyloid fibrils are larger and insoluble and can further aggregate into amyloid plaques, forming histopathological lesions, whereas amyloid oligomers are soluble, neurotoxic, and capable of propagating neuronal damage (Figure 3a) [29]. In addition, sAPPβ can also bind and interact with numerous molecules in the extracellular space, such as cell surface receptors, metals, and lipid membranes, thereby activating downstream signaling pathways that generate reactive oxygen species, promote tau hyperphosphorylation, and induce inflammatory responses that ultimately result in neuronal death and contribute to AD.

Aβ amyloid plaques and abnormal neurites are classified according to their relative amount, distribution, and morphology into: (1) diffuse or pre-amyloid plaques, which consist of amorphous amyloid deposits with undefined boundaries, loosely arranged Aβ filaments, and a lack of dystrophic neurites; Aβ1-42 is the principal constituent of these plaques; (2) non-cored neuritic plaques, which exhibit distinctive pores and structural irregularities. They are oval or spherical formations containing Aβ and altered neurites and lack a densely packed Aβ region in the central portion; (3) dense core neuritic plaques, which consist of compact nuclei surrounded by loose fibrillar deposits and predominantly containing Aβ1-42. They are found primarily in regions such as the hippocampus and cerebral cortex and increase markedly with age. Nearby are tau-positive dystrophic neurites, reactive astrocytes, and activated microglia. Furthermore, given their association with neuronal loss and cognitive decline, these plaques represent a hallmark for the diagnosis of AD; and (4) compact or burnt-out plaques, which are characterized by a dense core lacking a surrounding neuritic component and are primarily constituted by Aβ1-40 [11]. Recently, 81 mammalian orthologous genes that enhance Aβ1-42 toxicity and 157 that have a protective effect have been identified [30].

In AD, the amyloidogenic pathway can be favored by several factors, including mutations in the *APP* and *PSEN* genes, overexpression of APP or β-secretases, inflammation, oxidative stress, environmental factors, interactions between tau and APP, and impaired Aβ clearance mechanisms [23]. For instance, mutations in *APP* lead to increased Aβ synthesis, while mutations in the transmembrane region of *APP* alter the Aβ1-42/Aβ1-40 ratio, particularly in the EOAD. Examples of *APP* genetic variants associated with increased Aβ production, enhanced Aβ oligomerization, and increased Aβ fibril formation include D678H (Taiwanese), E693G (Arctic), and E682K (Leuven) [31]. Higher levels of Aβ production may also result from mutations in *PSEN1* and *PSEN2*, with *PSEN1* mutations specifically leading to increased Aβ1-42 formation [32].

Importantly, Aβ deposition induces tau pathology by activating kinases such as GSKβ and CDK that mediate hyperphosphorylation, and hyperphosphorylated tau mediates neuronal toxicity. GSKβ activity is also increased by the *APOE* ε4 allele, thereby contributing to tau pathology [33]. In turn, the presence of tau modulates and influences Aβ cytotoxicity. Hence, Aβ and tau act synergistically on cellular processes and organelles, amplifying each other’s toxic effects. They may interact through intermediate molecules such as GSK-3β, CDK-5 and ERK. Furthermore, Aβ and tau can coexist at pathological sites: extracellular deposits of fibrillar Aβ form senile plaques and accelerate tau hyperphosphorylation, which in turn induces neurotoxicity [34].

Current therapeutic approaches, such as BACE inhibitors and γ-secretase inhibitors/modulators are able to slow the production of β-amyloid, which represents the initial phase of the amyloid cascade. However, they are unable to eliminate existing Aβ plaques and therefore cannot ameliorate the toxic events already triggered by these Aβ aggregates. Furthermore, Aβ accumulation stimulates tau protein phosphorylation and the formation of NFTs, leading to neurodegeneration.

Therefore, the use of tau kinase inhibitors that prevent phosphorylation or compounds that inhibit tau protein aggregation and/or promote the disintegration of aggregates may be beneficial [24].

### 2.2. Neurofibrillary Tangles Structure and Functions

In AD, tau protein becomes abnormally hyperphosphorylated (p-tau), leading to its aggregation into neurofibrillary tangles (NFTs) within neurons. Typically, NFTs first appear in the allocortex of the medial temporal lobe (entorhinal cortex and hippocampus), then spreads to the association isocortex [19], and ultimately to the neocortex [13]. As such, NFTs represent hallmark pathological features of both AD and other neurodegenerative disorders, also known as tauopathies, including Pick’s disease, frontotemporal dementia, corticobasal degeneration, argyrophilic grain disease, progressive supranuclear palsy, and Huntington’s disease [35]. There are also cases classified as primary age-related tauopathy or “primary age-related tauopathy” (PART) in which NFTs are found in the brains of elderly individuals who may show no cognitive impairment deterioration or only mild amnestic changes, even in the absence of amyloid plaques [2].

Tau is one of the main microtubule-associated proteins (MAPs) present in the cytosol and the axon of neurons of normal brains, although it may also be found in the soma and dendrites [23,36]. Its main function is to promote tubulin polymerization through the regulation of phosphorylation and to maintain microtubule structure, stability, and assembly [37]. Its hydrophilic structure consists of four domains: (1) a N-terminal domain, which plays an important role in regulating the distance between microtubules; (2) a proline-rich region (PRR), which contributes to cell signaling and interaction with protein kinases and contains abundant phosphorylation sites; (3) a microtubule-binding region (MTBR), which contains three or four internal repeats that mediate binding to the proline-rich domain; and (4) a C-terminal domain, which is involved in microtubule polymerization. Different tau isoforms possess specific physiological properties and are differentially expressed during brain development [33,38].

Tau protein is encoded by the *MAPT* (microtubule-associated protein tau) gene, which is located on chromosome 17q21.31 and consists of 16 exons. This gene contains four types of repeated sequences: (1) short interspersed nuclear elements (SINEs), (2) long interspersed nuclear elements (LINEs), (3) DNA transposons, and (4) transposable elements with long terminal repeats (LTRs) [39]. Exons 2, 3, and 10 of the *MAPT* gene are expressed in the adult brain and are subject to alternative splicing, which plays a major role in the generation of six tau isoforms (3R0N, 3R1N, 3R2N, 4R0N, 4R1N, and 4R2N) that are differentially expressed during brain development. The fetal human brain expresses only the shortest isoform 3R0N [40]. Furthermore, alternative splicing of exon2 and exon3 produces variants that contain zero (0N), one (1N), or two (2N) N-terminal inserts. Exons 9–12 encoded some repeated domains (R1–R4) located at the C-terminus, through which tau protein binds to microtubules [41]. Tau can also be regulated by post-translation modifications (PTMs) that may promote aggregation, such as phosphorylation, dephosphorylation, isomerisation, glycation, nitration, addition of β-linked N-acetylglucosamine (O-GlcNAcylation), acetylation, oxidation, polyamination, sumoylation, ubiquitylation and truncation (proteolytic cleavage) at serine, threonine and tyrosine residues [40,42].

The major alterations of physiological tau are phosphorylation and dephosphorylation, which are influenced by specific protein kinases and phosphatases and may occur at 85 potential sites located on serine (S), threonine (T), and tyrosine (Y) residues [43]. In particular, phosphorylation sites have been identified in the N-terminal region (Ser46, Thr123, Ser198, Ser199, Ser202/205, Ser208, Ser210, Thr212, Ser214, Thr217, Thr231, and Ser235), the repeat region (Ser262 and Ser356), and the C-terminal region (Ser396, Ser400, Thr403, Ser404, Ser409, Ser412, Ser413, and Ser422) [34,44]. These sites are phosphorylated by several kinases, including protein kinase A (PKA), protein kinase C (PKC), cyclin-dependent kinase-5 (CDK-5), Ca^2+^/calmodulin-dependent kinase II (CaMKII), glycogen synthase kinase-3β (GSK-3β), and mitogen-activated protein kinase (MAPK). These kinases contribute to hyperphosphorylation of tau, leading to tau detachment from microtubules and the subsequent formation of neurofibrillary tangles [34].

Phosphorylated tau (P-tau) contributes to neurodegeneration by disrupting axonal transport and synaptic function, altering cellular stress response, and promoting neuroinflammation [45]. In its native state, tau is relatively stable and unlikely to misfold. However, its inherently disordered structure can gradually adopt a β-sheet conformation, increasing susceptibility to aggregation. Formation of β-pleated sheet structures strengthens interactions between p-tau monomers, promoting the assembly of larger aggregates [46]. Under pathological conditions, hyperphosphorylation of tau reduces its affinity for microtubules, leading to cytoskeletal destabilization [42].

In AD, highly phosphorylated tau is observed not only in axons but also in the cytosol and dendrites. Moreover, in this pathological context, tau can exist in different structural states, including soluble monomers, oligomers, and fibrillar aggregates [23]. Oligomers are formed when monomeric p-tau molecules associate through nucleation processes, with central units acting as structural cores. These oligomeric assemblies can progressively grow as additional p-tau monomers are recruited, eventually giving rise to larger aggregates. Such oligomers represent intermediate structures that precede the formation of fibrillary tangles (Figure 3b) [47].

To date, no *MAPT* mutations have been identified as causative of AD; however, several mutations in the *MAPT* gene have been reported to cause frontotemporal dementia [37]. Most known mutations in the *MAPT* gene are located in the C-terminal region, particularly within exons 9–12, which encode the microtubule-binding repeat domains. These mutations can alter the levels of tau isoforms and impair their ability to bind to and stabilize microtubules, thereby promoting tau aggregation into filaments. Some *MAPT* mutations also affect the ratio between 3R and 4R tau isoforms and may accelerate tau phosphorylation.

As stated above, several therapeutic strategies targeting tau are currently under development, including immunotherapies (antibodies and vaccines) as well as approaches aimed at suppressing tau synthesis (such as siRNA, miRNA and antisense oligonucleotides), inhibitors of tau aggregation (e.g., LMTX developed by TauRx Therapeutics), and compounds designed to regulate tau post-translational modifications [10].

### 2.3. Autophagy Dysregulation in Alzheimer’s Disease

Autophagy is an essential intracellular, self-degradative process that removes misfolded or accumulated proteins as well as defective or damaged organelles through lysosomal degradation, while recycling basic cellular components in eukaryotic cells to generate energy and maintain intracellular homeostasis. Through these mechanisms, autophagy promotes cell survival and suppresses programmed cell death [48,49]. This molecular pathway also plays a key role in the metabolism of Aβ, regulating both its generation and clearance [50]. Indeed, alterations in autophagy can lead to the accumulation of toxic proteins in the brain and contribute to the development of AD [51]. Moreover, impairment of this process negatively affects tau clearance and promotes tau pathology [52]. In the vicinity of extracellular Aβ plaques, brain macrophages, and therefore microglia are recruited and attempt to eliminate pathological Aβ deposits through phagocytosis. However, internalized Aβ can escape from the endolysosomal system into the cytosol, leading to cellular damage.

The interaction between microglia and Aβ plaques is facilitated by the expression of several Aβ receptors on microglia cells, including triggering receptor expressed on myeloid cells 2 (TREM2), low-density lipoprotein receptor-related protein 1 (LRP1), Toll-like receptors (TLRs), complement receptor 3 (CR3), CD14, CD47, CD36, integrins, receptor for advanced glycation end products (RAGE), and α6β1 integrin [2].

Under physiological conditions, autophagy is constitutively active and cells perform low levels of autophagy, also known as basal autophagy [44]. It can be rapidly induced by multiple stimuli, such as energy deprivation, nutrient starvation, misfolded proteins, damaged organelles, infection, inflammation, and other stressors [48]. During the progression of AD, aberrant autophagy occurs, resulting in the accumulation of mutated toxic proteins (e.g., Aβ and tau), which leads to increased oxidative stress and neuronal death [48]. In the early stages of the disease, the autophagy mechanism may be activated as a compensatory response to the accumulation of Aβ plaques and tau tangles in the attempt to eliminate toxic aggregates; however, the presence of aggregated proteins can hinder the autophagic process, generating a vicious cycle in which impaired autophagy causes further protein accumulation, thereby exacerbating neurodegeneration. Therefore, it has been reported that autophagic efficiency may decline as AD progresses [51].

There are four major types of autophagy: (1) macroautophagy, a conserved cellular process in eukaryotic cells responsible for the degradation and recycling of cytoplasmic components [53]. It represents the predominant form of autophagy and can be further subdivided into selective pathways, such as aggrephagy or mitophagy, depending on the targeted substrate [54]. During this process, double-membrane structures known as a phagophore form and engulf portions of the cytoplasm, including organelles, to give rise to autolysosomes in which the sequestered material is degraded. These vesicles subsequently fuse with lysosomes, forming autolysosomes following the fusion of the autophagosomal outer membrane with the lysosomal membrane; (2) chaperon-mediated autophagy (CMA), a selective pathway in which specific cargo is directly translocated across the lysosomal membrane without the involvement of vesicular intermediates. This process relies on the presence of a consensus pentapeptide motif (Lys-Phe-Glu-Arg-Gln; KFERQ) within substrate proteins. Cytosolic heat shock protein 70 (HSP70) recognizes and binds the substrate, forming a complex that is targeted to lysosome-associated membrane protein 2A (LAMP2A), which acts as the CMA receptor. The substrate is subsequently unfolded, translocated into the lysosomal lumen, and degraded; (3) microautophagy, the simplest form of autophagy, characterized by the direct engulfment of cytoplasmic material by the lysosome or vacuole through membrane invagination. This process may occur in a non-selective manner or selectively target specific substrates, such as peroxisomes (micropexophagy) [44,55]; (4) and mitophagy, a specialized form of selective autophagy that functions as a mitochondrial quality control mechanism by promoting the removal of damaged or dysfunctional mitochondria, thereby preserving cellular homeostasis [56]. This latter autophagic mechanism is closely linked to AD because neurons are highly dependent on healthy mitochondria for energy production and the regulation of cellular survival. In AD, however, mitophagy becomes impaired, leading to the accumulation of dysfunctional mitochondria within neurons.

In the AD brain, an excess of enlarged, poorly acidified autolysosomes and autophagosomes containing β-amyloid and tau has been observed. These structures accumulate and swell, forming large, distended, rosette-shaped, autophagosome-associated vesicle-like membranous protrusions surrounding the neuronal nucleus, known as panthos. These swollen autophagic structures appear to induce cell death, resulting in the release of intracellular amyloid into the extracellular environment, thereby contributes to the formation of amyloid plaques [53]. Furthermore, unlike soluble tau which is primarily degraded by the ubiquitin-proteasome system (UPS), oligomeric and hyperphosphorylated tau is preferentially degraded by autophagy. Hyperactivation of the mTORC1 complex, which inhibits autophagy, together with inhibition of the Unc-51-like kinase 1 complex (ULK1C), which promotes autophagy, has also been observed. Autophagosomes and autolysosomes contain APP and its processing enzymes and can serve as sites for the generation of β-amyloid and APP C-terminal fragments (CTFs). The accumulation of poorly acidified autolysosomes containing undigested material induces autophagic stress, and these vesicles can fuse with the plasma membrane, providing a pathway for the unconventional secretion of both β-amyloid and tau [53].

Mutations in genes implicated in AD may disrupt autophagy. *APP* mutations can induce defective autophagy and mitophagy, leading to increased generation of Aβ monomers and oligomers within lysosomes, while Aβ overload further impairs autophagy and lysosomal degradation. Mutations in *PSEN1* impair lysosomal enzymatic activity and autophagic protein degradation, as PSEN1 is required for lysosomal acidification. In contrast, *PSEN2* mutations disrupt autophagy by altering intracellular Ca^2+^ homeostasis [49]. *APOE4* can suppress autophagy by repressing FoxO3a signaling or by directly binding to coordinated lysosomal expression and regulation (CLEAR) motifs. APOE is also a ligand for triggering receptor expressed on myeloid cells 2 (TREM2) in microglia, and *TREM2* mutations (R47H or R62H) are associated with increased numbers of autophagic vesicles in AD patients compared with controls [49].

Sirtuin 5 (SIRT5) also modulates autophagy: its overexpression enhances autophagy and reduces neuroinflammation in AD-affected brains and neurons, whereas inhibition of autophagy abolishes the neuroprotective effects of SIRT5 [14].

### 2.4. Mitochondrial Reactive Oxygen Species (ROS) Production and Neurotoxic Signaling in Alzheimer’s Disease

Mitochondria are dynamic and essential organelles that perform multiple functions in cellular processes. They continuously undergo fission and fusion within eukaryotic cells [57] and are recognized as the powerhouse of the cell due to their role in ATP production through oxidative phosphorylation (OXPHOS), which is necessary to maintain neuronal homeostasis and function [58]. In the brain, their high energy output is primarily required for synaptic transmission, synaptogenesis, and synaptic activity [59].

Mitochondria also play a key role in buffering intracellular calcium (Ca^2+^), thereby regulating calcium levels during signal transduction, which is particularly important in excitable cells such as neurons [60]. Ca^2+^ influx across the outer mitochondrial membrane is mediated by voltage-dependent anion channel 1 (VDAC1), while transport into the matrix is regulated by the mitochondrial calcium uniporter (MCU) complex, including EMRE, MICU1, MICU2, and MCUR1 [59]. Another critical function is the regulation of programmed cell death (apoptosis), mediated by the release of proteins such as cytochrome c or mitochondrial DNA (mtDNA) from the mitochondrial intermembrane space into the cytosol in response to intrinsic signals [61,62].

**Figure 4:**
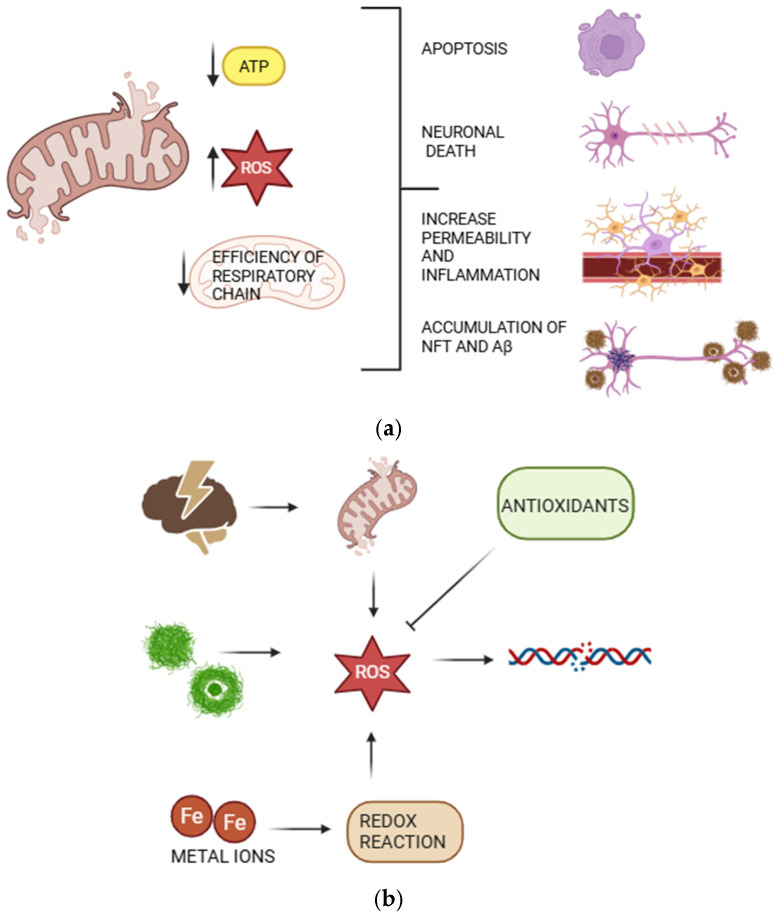
(**a**) Mitochondrial dysfunction in AD: mitochondria exhibit impaired bioenergetic function, including reduced ATP production and deficits in oxidative phosphorylation, accompanied by increased generation of reactive oxygen species (ROS). These alterations lead to increased mitochondrial membrane permeability, activation of apoptotic pathways, neuronal death, and neuroinflammation. Mitochondrial dysfunction also contributes to the accumulation of Aβ and the formation of neurofibrillary tangles, further promoting synaptic failure and neurodegeneration. (**b**) OS in AD: excessive ROS/RNS production overwhelms neuronal antioxidants (SOD, catalase, glutathione). Dysregulated metal ions (Fe, Cu, Zn) catalyze ROS formation, leading to lipid, protein, and DNA damage. This promotes Aβ accumulation, tau hyperphosphorylation, mitochondrial dysfunction, neuroinflammation, and neuronal death.

In AD, mitochondrial dysfunction, including alteration in mitochondrial morphology and function, is considered an early pathogenetic event that impairs neuronal function and viability, thereby promoting early neuronal death and the onset of disease symptoms [61]. Indeed, mitochondrial dysfunction underlies several pathophysiological changes that are observed in AD brains, including reduced metabolism, disruption of Ca^2+^ homeostasis, increased oxidative stress, elevated ROS production, lipid peroxidation, and apoptosis (Figure 4a) [62]. Moreover, the mitophagy impairment occurring in AD may prevent the timely clearance of damaged mitochondria [61] leading to the accumulation of dysfunctional mitochondria, and further exacerbating cellular stress and creating a detrimental feedback loop [63].

Mitochondrial dysfunction may result from the Aβ intracellular accumulation within mitochondria that disrupts their structure and impairs their respiratory function and ATP production, while increasing mitochondrial-associated oxidative stress. Thus, as mitochondria are the primary intracellular source of reactive oxygen species (ROS) [64], Aβ peptides further exacerbate ROS production. Elevated mitochondrial ROS promotes the release of cytochrome c and apoptosis-inducing factor, leading to mitochondrial dysfunction, activation of apoptotic signaling pathways, and subsequent neuronal loss. Importantly, age- and disease-related increases in ROS arising from impaired mitochondrial function, disrupted metal homeostasis, and weakened antioxidant defenses, contribute to synaptic dysfunction, impaired neurotransmission, and progressive cognitive decline.

The mitochondrial protein apoptosin also plays a crucial role in Aβ-induced neuronal death, as reduced apoptosin expression confers protection against Aβ-mediated neurotoxicity. Furthermore, other mitochondrial proteins, including amyloid-binding alcohol dehydrogenase and cyclophilin D, have been implicated in Aβ-related mitochondrial dysfunction [5].

When mitochondrial dysfunction occurs, it may also promote tau aggregation through cytochrome c release and subsequent activation of caspase-3, which enhances tau cleavage, aggregation, and hyperphosphorylation, ultimately contributing to the formation of neurofibrillary tangles (NFTs). Pro-apoptotic signals induce BCL2 family-mediated pore formation in the mitochondrial outer membrane, leading to cytochrome c release and caspase activation. Concurrently, mtDNA released into the cytosol acts as a damage-associated molecular pattern (DAMP), activating the cyclic GMP–AMP synthase–stimulator of interferon genes (cGAS–STING) pathway, an innate immune signaling cascade that detects cytosolic DNA and triggers type I interferon responses, thereby promoting neuroinflammation. Accumulation of mtDNA mutations over time further exacerbates mitochondrial dysfunction and oxidative stress, thereby enhancing apoptosis [65].

Deficiencies in mitochondrial metabolic enzymes, respiratory chain dysfunction, and reduced the cytochrome oxidase activity in the frontal, temporal, and parietal cortices have been reported in AD patients [66]. Excess ROS resulting from mitochondrial respiratory chain dysfunction inhibits protein phosphatase 2A (PP2A), the major tau dephosphorylase, and activates glycogen synthase kinase-3β (GSK-3β), a key tau kinase. This imbalance leads to tau hyperphosphorylation and the formation of neurofibrillary tangles, a hallmark of AD [65]. Moreover, elevated ROS levels stimulate pro-inflammatory gene transcription and the release of cytokines such as interleukin (IL)-1, IL-6, and tumor necrosis factor-α (TNF-α), as well as chemokines, thereby promoting neuroinflammation and neuronal damage [67,68]. Based on this evidence, mitochondria represent a promising therapeutic target, although their double-membrane structure poses challenges for drug delivery. Current strategies under investigation include mitochondria-targeted antioxidants such as MitoQ [61,63], mitophagy enhancers targeting the PINK/Parkin pathway, nanostructured lipid carriers to improve blood–brain barrier penetration and targeted delivery, and inhibitors of the mitochondrial permeability transition pore (mPTP), such as cyclosporine A [61].

### 2.5. Role of Oxidative Stress in Neuronal Damage and Alzheimer’s Disease Pathogenesis

Oxidative stress (OS) represents a central downstream consequence of mitochondrial dysfunction in AD and plays a key role in amplifying neuronal damage and neurodegeneration. This condition arises from an age- and disease-related imbalance between reactive oxygen species (ROS) production and antioxidant defenses, directly impairing synaptic function, disrupting neuronal signaling, and ultimately contributing to cognitive decline. In the early stages of AD, increased levels of oxidized proteins have been observed, particularly in Aβ-rich brain regions, such as the hippocampus and cortex [14].

OS is defined as “an imbalance in pro-oxidants and antioxidants in the body with associated disruption of redox circuitry and macromolecular damage”, and is characterized by increased production of ROS and reactive nitrogen species (RNS) including superoxide radical anion (O^2–^), hydrogen peroxide (H_2_O_2_), hydroxyl radical (HO^–^), nitric oxide (NO), and peroxynitrite (ONOO^–^) [64]. Thus, OS occurs when ROS production exceeds the capacity of cellular antioxidant defense system [69].

Antioxidants are essential for protecting the brain from ROS-induced damage and can be classified into two main categories: (1) enzymatic antioxidants, which are protein or metalloproteins, including superoxide dismutase (SOD), catalase (CAT), glutathione peroxidase (GSH-Px), and methionine sulfoxide reductase (MSR); and (2) non-enzymatic antioxidants, such as vitamin C, vitamin E, carotenoids, uric acid, ubiquinone, and ferritin [70].

Excessive OS can trigger cell death by disrupting physiological pathways, particularly those involving calcium (Ca^2+^) signaling. Oxidative conditions promote Ca^2+^ influx into the cytosol from both the extracellular space and intracellular stores, such as the endoplasmic and sarcoplasmic reticulum (ER/SR), through plasma membrane channels. The resulting elevation in cytosolic Ca^2+^ further drives its accumulation in the nucleus and mitochondria, ultimately leading to cell death [71].

Increased ROS also enhance the production, misfolding, and aggregation of Aβ, including the formation of cross-linked species. Aβ peptides contain a sequence in which the methionine residue at position 35 is particularly susceptible to oxidative modification. Under normal physiological conditions, ROS can oxidize methionine residues, leading to the formation of oxidized methionine species. In contrast, in AD, increased oxidative stress results in a greater accumulation of these oxidized species, which have been detected in affected brain regions [69].

Metal ions, particularly copper (Cu), iron (Fe), and manganese (Mn), are key contributors to OS, as they participate in both ROS generation and antioxidant defense mechanisms [72]. Amyloid plaques, composed of Aβ aggregates, are enriched in metal ions such as Zn, Cu, and Fe. Aβ oligomers can bind these transition metals, accelerating peptide aggregation and further promoting oxidative reactions. In turn, Aβ aggregation generates ROS that react with proteins and lipids, leading to the formation of oxidized proteins and lipid peroxides, thereby amplifying cellular damage.

To counteract OS, neurons, astrocytes, and microglia activate endogenous antioxidant defenses primarily regulated by nuclear factor erythroid 2–related factor 2 (Nrf2). As a master transcription factor, Nrf2 controls the expression of approximately 250 antioxidant response element (ARE)-containing genes involved in metabolism, inflammation, and antioxidant responses. Elevated ROS levels induce Nrf2 translocation into the nucleus, where it binds to AREs and activates antioxidant defense pathways [71].

Persistent ROS overproduction disrupts the balance between oxidant generation and antioxidant defenses, overwhelming the cellular detoxification capacity and leading to oxidative damage, mitochondrial dysfunction, and neuronal injury (Figure 4b) [57]. Oxidative modifications alter protein conformation, causing loss of function and promoting aggregation through hydrophobic interactions, ultimately contributing to cell death. Aβ42 and Aβ40 are highly hydrophobic peptides with a redox-active methionine residue at position 35, which contributes to their susceptibility to oxidative damage [73].

In the early stages of AD, activation of the unfolded protein response (UPR) protects neurons by reducing the burden of misfolded proteins and limiting their abnormal accumulation. However, prolonged cellular stress leads to sustained UPR activation, which can trigger apoptotic signaling and contribute to endoplasmic reticulum (ER) stress. Moreover, UPR efficiency declines with age. PSEN1 is involved in the activation of inositol-requiring enzyme 1 (IRE1), a transmembrane kinase that plays a key role in UPR signaling. In AD, disruption of UPR initiation impairs the clearance of misfolded proteins, promoting their accumulation, exacerbating ER stress, and ultimately leading to neurodegeneration and neuronal death. ER stress can also activate inflammatory pathways and may contribute to autoimmune responses associated with defective protein folding [74]. Overall, OS contributes to the amplification of neurodegeneration process by promoting the formation and accumulation of Aβ and NFTs. Accordingly, targeting OS has emerged as a potential therapeutic strategy, with antioxidant-based approaches aimed at restoring redox homeostasis and mitigating oxidative damage through dietary and pharmacological interventions [33].

### 2.6. Neuroinflammation in Alzheimer’s Disease

Closely linked to OS, neuroinflammation, triggered by microglia and astrocytes, represents another critical pathological mechanism contributing to the development and progression of AD. Although microglia play a fundamental role in the brain under physiological conditions, increasing evidence indicates that the chronic activation of microglia contributes to neuronal dysfunction and accelerates neurodegeneration. It is well established that the accumulation of pathological protein aggregates typical of AD, including Aβ plaques and NFTs composed of hyperphosphorylated tau, strongly stimulates immune cells of the central nervous system (CNS). These protein aggregates act as damage-associated molecular patterns (DAMPs) that chronically activate microglia [75]. Indeed, the interaction between pattern recognition receptors (PRRs), including Toll-like receptor 2 (TLR2) and Toll-like receptor 4 (TLR4), membrane-bound receptors that recognize pathogen and damage-associated molecular patterns and the NLRP3 inflammasome (NOD-like receptor family pyrin domain containing 3), a cytosolic multiprotein complex involved in innate immune activation, and DAMPs induces a shift toward a pro-inflammatory phenotype, leading to the release of inflammatory mediators such as cytokines including interleukin-1β (IL-1β), interleukin-6 (IL-6), and tumor necrosis factor-α (TNF-α) as well as chemokines, and to the production of reactive oxygen species (ROS) and nitric oxide (NO). This initially protective response becomes deleterious when prolonged over time, resulting in sustained neuroinflammation that exacerbates neuronal damage [76].

**Figure 5:**
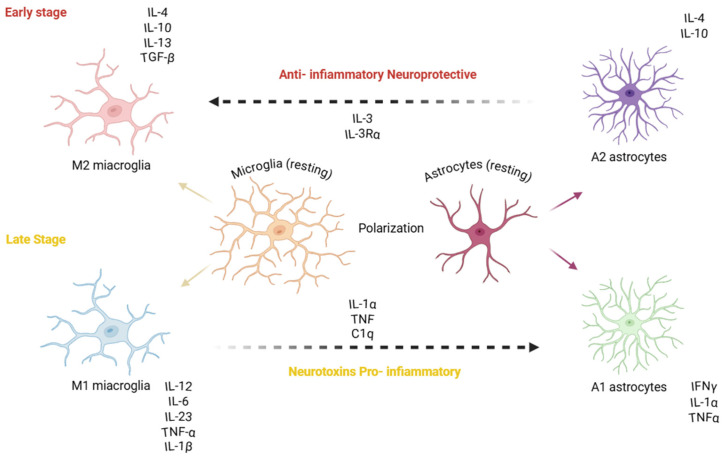
Neuroinflammation process: Aβ plaques and hyperphosphorylated tau activate microglia and astrocytes. Activated glia release pro-inflammatory (M1/A1), neurotoxic molecules (e.g., TNF-α, IL-1β, IL-6, ROS/RNS) that promote neuronal stress, synaptic dysfunction, and cell death. At the same time, glial cells can produce anti-inflammatory (M2/A2) and neuroprotective factors (e.g., IL-10, TGF-β, neurotrophic factors) that counteract damage. A chronic imbalance favoring pro-inflammatory, neurotoxic signaling exacerbates Aβ and tau pathology, driving neurodegeneration.

Astrocytes also play a key role in the neuroinflammatory response associated with AD. When exposed to inflammatory signals released by activated microglia and Aβ deposits, astrocytes undergo a process known as reactive astrogliosis (Figure 5) [77]. Consequently, their normal neuroprotective functions including regulation of neurotransmitter balance, metabolic support to neurons, and maintenance of the blood–brain barrier become compromised. Moreover, by producing complement proteins, additional cytokines, and other inflammatory mediators, astrocytes further amplify immune signaling within the brain.

Additionally, a close interconnection between OS and neuroinflammation has been reported, with these processes reinforcing each other during AD progression. Elevated ROS levels can activate inflammatory signaling pathways, including nuclear factor kappa B (NF-κB), which promotes the expression of pro-inflammatory genes. Conversely, inflammatory cells generate additional ROS and reactive nitrogen species, thereby intensifying oxidative damage and creating a vicious cycle that accelerates neuronal injury [78].

Recent studies have provided important insights into the mechanisms underlying neuroinflammation in AD, highlighting the contribution of immune-related genes to disease susceptibility. Genome-wide association studies (GWAS) have identified several susceptibility loci linked to genes involved in innate immunity, modulation of microglial activation and function, inflammatory signalling pathways and phagocytosis. Among theme, variants in the *TREM2*, *CD33*, *CR1*, and *ABCA7* genes have been identified [79,80].

In particular, rare variants in *TREM2* have been shown to impair microglial responses to amyloid-β accumulation, reducing plaque clearance and promoting a self-sustaining inflammatory environment that exacerbates synaptic dysfunction and neuronal loss [81].

Overall, chronic neuroinflammation contributes to synaptic dysfunction, neuronal loss, and cognitive decline in AD. Consequently, therapeutic strategies aimed at modulating microglial activation, reducing inflammatory signaling, and restoring immune homeostasis are being actively investigated as potential approaches to slow or prevent disease progression [82].

### 2.7. Necroptosis in Alzheimer’s Disease

Recent evidence from neuropathological and experimental studies consistently supports the view that necroptosis contributes to neuronal loss and neuroinflammation in AD. Necroptosis is a regulated form of necrotic cell death that is mediated by the activation of receptor-interacting serine/threonine-protein kinase 1 (RIPK1) and receptor-interacting serine/threonine-protein kinase 3 (RIPK3), both key regulators of programmed necrosis involved in inflammatory signaling pathways which phosphorylate the mixed lineage kinase domain-like protein (MLKL), leading to its translocation to the plasma membrane, cell lysis, and the release of intracellular pro-inflammatory contents [83,84]. Indeed, post-mortem analyses of AD brains reveal that elevated levels of phosphorylated RIPK1, RIPK3, and MLKL correlate with disease severity and cognitive impairment [85].

Additionally, in mouse models of AD, inhibition of necroptotic signaling via pharmacological or genetic approaches reduces neuronal loss and mitigates memory deficits, suggesting a causal role for necroptosis in AD pathology [86].

Necroptosis also appears to intersect with key pathogenic mechanisms in AD. The accumulation of Aβ oligomers is not only toxic to neurons but also acts as a potent trigger of necroptotic signaling. Indeed, Aβ can activate microglial cells, leading to the release of inflammatory cytokines such as TNF-α, which in turn stimulates tumor necrosis factor receptor 1 (TNFR1) on neurons. This activation promotes the recruitment and phosphorylation of RIPK1, initiating the necroptotic cascade. Subsequently, RIPK3 is activated and phosphorylates MLKL, which translocates to the plasma membrane and disrupts its integrity. As a result, neurons undergo lytic cell death. This process amplifies neuroinflammation and creates a vicious cycle of inflammation and cell death [85]. The disruption of cytoskeletal organization and the impairment intracellular transport caused by tau hyperphosphorylation lead to mitochondrial dysfunction, increased OS, and energy deficits, all of which sensitize neurons to necroptotic signaling.

Tau pathology is closely linked to necroptotic mechanisms. By promoting mitochondrial dysfunction, increased OS, and energy deficits, it further sensitizes neurons to necroptotic signaling [85]. Furthermore, the interaction between tau pathology and necroptosis is likely bidirectional. In particular, necroptosis can exacerbate tau-related alterations, as the inflammatory milieu generated by necroptotic cell death promotes the activation of kinases responsible for tau phosphorylation, ultimately enhancing tau aggregation.

Additionally, neuroinflammation plays a central role in linking Aβ, tau, and necroptosis. Activated microglia and astrocytes produce pro-inflammatory mediators that sustain necroptotic signaling, particularly through chronic activation of RIPK1. In this context, necroptosis acts both as a driver and as a consequence of inflammation, forming a self-perpetuating loop that accelerates neurodegeneration. Given its central role as a mechanistic hub linking β-amyloid toxicity, tau pathology, and neuroinflammation, necroptosis represents a particularly promising therapeutic target for slowing disease progression [84].

### 2.8. Blood–Brain Barrier (BBB) Dysfunction and Vascular Contributions in Alzheimer’s Disease

Increasing evidence suggest that vascular dysfunction and alterations of the neurovascular unit play a fundamental role in the initiation and progression of AD. Over the last two decades, a large body of experimental and clinical studies has demonstrated that cerebrovascular alterations including cerebral hypoperfusion, endothelial dysfunction, pericyte degeneration, and breakdown of the blood–brain barrier (BBB) are early features of AD that can precede the onset of classical neuropathological changes and cognitive symptoms [87]. In this context, the two-hit vascular hypothesis of AD provides an integrative model linking vascular pathology with amyloid accumulation and neurodegeneration. According to this hypothesis, vascular dysfunction represents the initial insult that perturbs brain homeostasis and promotes the accumulation of amyloid-β, which in turn amplifies neuronal injury and neurodegeneration and further damages the cerebrovascular system [88]. Central to this process is the progressive disruption of the BBB, a highly specialized interface responsible for maintaining the biochemical and cellular homeostasis of the CNS. This interface consists of several cell types that collectively form the neurovascular unit (NVU), including pericytes embedded within the capillary basement membrane, brain endothelial cells, astrocytic end-feet, neurons, and extracellular matrix components. One of the key physiological processes mediated by the NVU is neurovascular coupling, a mechanism through which local neuronal activity leads to rapid adjustments in cerebral blood flow [88]. In response to neuronal activation, astrocytes and endothelial cells release vasoactive mediators such as nitric oxide and prostaglandins, causing dilation of nearby blood vessels and increased perfusion [89]. In AD, however, the structural and functional integrity of the neurovascular unit becomes progressively compromised. Endothelial cells exhibit altered signaling pathways, pericytes undergo degeneration, and astrocytes develop reactive phenotypes. These changes collectively impair neurovascular coupling and contribute to the development of chronic cerebral hypoperfusion [90]. This condition constitutes the first “hit” in the two-hit vascular hypothesis [88]. Aging, genetic susceptibility, and vascular risk factors such as hypertension, diabetes, dyslipidemia promote endothelial injury, OS, and inflammatory signaling in cerebral vessels, thereby contributing to this process [90].

The second pathogenic event in the two-hit model involves the impaired clearance and accumulation of Aβ. Given that the BBB plays a central role in Aβ clearance through specialized transport receptors, vascular abnormalities induced during the first hit exacerbate Aβ dysregulation, reducing its efflux from the brain and increasing its influx from the circulation, ultimately promoting amyloid accumulation within the brain parenchyma [91].

Among these transport receptors, low-density lipoprotein receptor-related protein 1 (LRP1) mediates the efflux of Aβ from the brain across the BBB, whereas the receptor for advanced glycation end products (RAGE) facilitates the influx of circulating Aβ into the brain. Dysregulation of these pathways contributes significantly to amyloid accumulation in AD [91].

Additionally, genetic risk factors, most notably the *APOE4* allele, exacerbate this imbalance by impairing LRP1-mediated clearance and promoting vascular inflammation, OS, and pericyte loss, thereby amplifying neurovascular dysfunction [92].

Indeed, *APOE4* carriers appear particularly vulnerable to these vascular and parenchymal amyloid-related changes, which may partly explain the increased risk and earlier onset of cognitive decline in genetically susceptible individuals [93].

The interaction between vascular dysfunction and amyloid pathology is further amplified by chronic inflammation and OS. When the BBB becomes permeable, circulating inflammatory mediators and immune cells can infiltrate the brain, activating resident microglia and astrocytes. The release of pro-inflammatory cytokines from activated glial cells exacerbates neuronal injury and further weakens vascular integrity [94].

At the same time, both endothelial cells and neurons exposed to Aβ produce increased levels of ROS a condition that exacerbates endothelial damage, promotes further BBB disruption, and accelerates neurodegeneration [95]. Given the critical role of neurovascular dysfunction in AD, increasing attention is being directed toward approaches that preserve or restore the integrity of the neurovascular unit. Potential therapeutic strategies include the stabilization of endothelial cells, protection of pericyte function, and modulation of inflammatory pathways that contribute to BBB disruption. Furthermore, enhancing Aβ clearance mechanisms, particularly those involving LRP1-mediated transport, represents a promising avenue for limiting amyloid accumulation [96].

## 3. Pharmacological Strategies in Alzheimer’s Disease

Therapeutic strategies are essential for managing symptoms, slowing disease progression, and improving quality of life in individuals with AD. Current approaches include both pharmacological and non-pharmacological interventions (such as cognitive training, physical exercise, and nutritional interventions). Among pharmacological options, therapies approved by the U.S. Food and Drug Administration (FDA) including cholinesterase inhibitors and N-methyl-D-aspartate (NMDA) receptor antagonists are the most widely used [33].

Being cognitive impairment in AD largely associated with the loss of cholinergic neurons, Acetylcholinesterase (AChE) inhibitors prevent the breakdown of acetylcholine, thereby increasing its levels in the synaptic cleft and enhancing neurotransmission. Drugs such as rivastigmine, donepezil, tacrine, and galantamine are commonly prescribed for mild to moderate AD [24,33].

Proteins involved in the amyloidogenic pathway represent important therapeutic targets due to their central role in amyloidogenesis. In particular, BACE1 is considered a key target [33], and the development of compounds capable of modulating its expression or inhibiting its enzymatic activity has emerged as a major pharmacological strategy to reduce Aβ production.

Huperzine A (Hup A), a natural neuroprotective alkaloid, has been shown to modulate APP metabolism by reducing BACE1 expression and PSEN1 levels while increasing ADAM10 (a disintegrin and metalloproteinase 10), ultimately leading to a significant decrease in Aβ accumulation [97]. In addition, M1 muscarinic receptor activity plays a crucial role in APP processing and tau phosphorylation, and its loss is associated with increased Aβ oligomer formation. Cotinine, a metabolite of nicotine, may also enhance cognitive function through modulation of α7 nicotinic acetylcholine receptors.

Lin CY et al. demonstrated that gliadin-derived protein hydrolysates, particularly bromelain-hydrolyzed gliadin (G-Bro), can inhibit BACE1 activity, reduce soluble APP levels, and prevent Aβ aggregation. This effect is attributed to specific peptide properties: proline disrupts β-sheet formation, lysine provides positive charges, hydrophobic residues stabilize binding interactions, and glutamine enhances solubility. Together, these features contribute to a mechanism that interferes with Aβ assembly, suggesting that G-Bro may represent a promising therapeutic candidate for AD [98].

Consistently, BACE1 inhibition has emerged as a major therapeutic strategy to reduce Aβ production. In this context, five compounds fluphenazine, naratriptan, bazedoxifene, frovatriptan, and raloxifene have been identified as potential BACE1 inhibitors. These molecules exhibit characteristic pharmacophoric features, such as indole or thioindole scaffolds, and establish stabilizing interactions with key residues in the enzyme’s active site, supporting their potential as lead compounds for AD drug development [99].

In recent years, the U.S. Food and Drug Administration (FDA) has approved several anti-Aβ monoclonal antibodies for the treatment of AD, aimed at reducing amyloid plaque burden in the brain (Table 1). Aducanumab (Aduhelm) was approved by the FDA in June 2021 for the treatment of mild cognitive impairment (MCI) or mild dementia, based on its ability to reduce Aβ plaques [100]. Although its marketing was discontinued in November 2024, it represented a milestone in targeted therapy for AD. Its mechanism of action involves selective binding to aggregated forms of Aβ, promoting their clearance.

Similarly, Lecanemab (Leqembi), an intravenous antibody infusion therapy that targets and removes Aβ from the brain, was approved by the FDA in January 2023 [101]. It binds soluble Aβ protofibrils, thereby reducing plaque accumulation and slowing disease progression [100].

Donanemab (Kisunla), another anti-Aβ antibody administered as an intravenous infusion every four weeks, targets the N-terminally truncated, pyroglutamate-modified forms of Aβ (N3pG) present in mature plaques, facilitating their removal [102,103]. It was approved by the FDA in July 2024 for the treatment of patients with MCI or mild dementia due to AD [104]. However, as with other Aβ-targeting antibodies, the risk of Amyloid-Related Imaging Abnormalities (ARIA) in patients treated with donanemab is dose-dependent and increases with the number of apolipoprotein E ε4 (*APOE4*) alleles. *APOE4* carriers are also more likely to experience recurrent, symptomatic, and severe ARIA, and the FDA has issued a boxed warning for the use of donanemab in *APOE4* homozygotes. Accordingly, *APOE* genotyping should be conducted before initiating treatment to assess an individual’s risk of developing ARIA [103].

Amyloid-related imaging abnormalities (ARIA), including edema (ARIA-E) and microhemorrhages or hemosiderin deposition (ARIA-H), represent only one of the adverse effects associated with anti-Aβ monoclonal antibodies. Therefore, although these therapies represent a significant advancement in the treatment of AD and offer hope for slowing disease progression, their clinical benefits, optimal use, and safety profiles remain under active investigation and debate [102].

**Table 1:** The principal clinical trials anti-amyloid-β monoclonal antibodies for the treatment AD. Data from clinicaltrials.gov (accessed on 13 April 2026).

Number Identifier	Study	Drugs	Data/Status	Outcomes	Main Side Effects
NCT05026866 [105]	Interventional	Donanemab	2027-11 (in progress)	Slows disease progression; prolongs participation in daily activities and independent living [106]	Serious allergic reactions, amyloid-related imaging abnormalities (ARIA) and headache [103,106]
NCT01148498 [107]		Solanezumab	2012-08 (completed)	No effect on cognitive decline; unsuccessful in stopping or slowing amyloid accumulation [108]	Data not provided
NCT03887455 [109]		Lecanemab	2029-06 (in progress)	Slows disease progression; supports prolonged independence in daily activities [110]	Serious allergic reactions, ARIA and headache [110]
NCT06285448 [111]	Observational	Lecanemab	2028-06 (in progress)	Results not provided	Data not provided
NCT04374253 [112]	Interventional	Gantenerumab	2023-03 (completed) 2023-03 (completed)	No association between monitoring and clinical decline; no long-term cognitive/functional effects	Suicidal ideation or suicidal behavior, ARIA-E, ARIA-H
NCT00667810 [113]	Interventional	Bapineuzumab	2013-08 (completed)	Brain amyloid positivity not an inclusion criterion; impact on cognitive and functional outcomes cannot be evaluated.	ARIA-E
NCT02353598 [114]		Crenezumab	2019-03 (completed)	safety, tolerability, and pharmacokinetics	AEs balanced across system organ classes; mostly low-grade, non-serious
**NCT04241068** **[115]**		**Aducanumab**	**2024-07 (completed)**	**Safety and tolerability [115]. Reduces cognitive and functional decline [116].**	**ARIA-E, ARIA-H, ADAs (positive antidrug antibodies)**

## 4. Conclusions and Future Perspectives

Genetic predisposition, aging, and environmental factors act together to drive gradual cognitive decline in AD, a complex and multifactorial neurodegenerative disorder. Although extracellular Aβ plaques and intracellular neurofibrillary tangles composed of hyperphosphorylated tau are the hallmark features of AD, they are part of a broader network of interconnected molecular pathways, including OS, mitochondrial dysfunction, defective autophagy, neuroinflammation, and dysregulated stress-response pathways. Furthermore, the high prevalence of sporadic late-onset AD underscores the importance of gene–environment interactions, particularly those involving the *APOE* ε4 allele, as well as age-related susceptibility. A deeper understanding of these mechanisms is essential to enable the development of reliable and accessible biomarkers capable of slowing disease progression and improving patient outcomes.

Building on these considerations, it becomes evident that addressing the complexity of AD requires moving beyond descriptive frameworks toward integrative and forward-looking research strategies.

The persistent failure of disease-modifying therapies in AD strongly suggests that reductionist, single-target strategies, primarily focused on amyloid-β or tau, are insufficient to halt disease progression. AD should instead be conceptualized as a systems-level disorder arising from the progressive disruption of interconnected molecular and cellular networks.

Future research should therefore prioritize integrative and multi-target approaches capable of simultaneously modulating key pathological processes, including neuroinflammation, mitochondrial dysfunction, impaired proteostasis, and dysregulated cell death pathways such as necroptosis. In this context, therapeutic strategies aimed at restoring network homeostasis, rather than targeting isolated molecular events, may represent a more effective paradigm.

Another critical challenge lies in the identification of early, sensitive, and accessible biomarkers that reflect the dynamic and preclinical stages of the disease. The integration of genetic risk factors, particularly *APOE* ε4, with longitudinal molecular and imaging data may enable improved patient stratification and facilitate the development of precision medicine approaches.

In addition, increasing evidence underlines the central role of non-neuronal components, including microglia, astrocytes, and the neurovascular unit. Blood–brain barrier dysfunction and cell type-specific interactions are emerging as key contributors to disease onset and progression, offering novel and still underexplored therapeutic targets.

Finally, a comprehensive understanding of gene–environment interactions and age-related vulnerability will be essential to fully capture the heterogeneity of AD. Moving forward, the integration of multi-omics data, advanced computational modeling, and longitudinal clinical studies will be crucial to translate molecular insights into effective and personalized interventions.

Collectively, these perspectives support a shift toward a more holistic and systems-oriented view of AD, which may ultimately enable the development of more effective therapeutic strategies and improve clinical outcomes.

## Data Availability

No new data were created or analyzed in this study. Data sharing is not applicable to this article.
